# Cheeklift With and Without Posterior Lamellar Spacer Grafts for Treatment of Lower Eyelid Retraction

**DOI:** 10.1007/s00266-024-03950-1

**Published:** 2024-03-18

**Authors:** Christie K. Campla, Caroline Awh, Nicole P. Rebollo, Julian D. Perry

**Affiliations:** 1 Cleveland Clinic Lerner College of Medicine, Cleveland, OH USA; 2 Cole Eye Institute, Cleveland Clinic Foundation, Cleveland, OH USA

**Keywords:** Eyelid retraction, Cheeklift, Spacer grafts

## Abstract

**Background:**

To compare outcomes of lower eyelid retraction repair using a subperiosteal midface lifting technique with and without posterior lamellar grafts.

**Methods:**

Charts of patients undergoing a sub-periosteal midface lift for treatment of lower eyelid retraction using 4 techniques for posterior lamellar reconstruction were reviewed. Thirty patients were included in each of the groups: midface with hard palate graft (HPG), midface lift with acellular cadaveric graft (ADG), midface lift with retractor disinsertion (RD) and midface lift alone (NG). Measurements of distance from pupil center to lower lid margin (MRD2) and from lateral limbus to lower lid margin (MRD2_limbus)_ were taken from pre- and postoperative photographs and compared. Secondary outcomes included rates of reoperation, major and minor complications, resolution of symptoms and keratopathy.

**Results:**

One hundred twenty operations were assessed (*n *= 30 for each surgical group). The average follow-up time was 20 weeks. The median MRD2 elevation was 0.95 mm (NG), 0.85 mm (HPG), 1.59 mm (ADG) and 1.02 mm (RD). The median MRD2_limbus_ elevation was 1.06 mm (NG), 0.92 mm (HPG), 1.45 mm (ADG) and 1.12 mm (RD). There were no significant differences in MRD2 or MRD2_limbus_ between the 4 groups (*p* = 0.06 and 0.29, respectively). Reoperation rates were highest with in the hard palate graft group (33%) compared to other techniques (*p *= 0.0006).

**Conclusions:**

Similar degrees of lower eyelid elevation were achieved with all the midface lifting techniques, and complication rates did not significantly differ between techniques. However, the higher reoperation rates with the use of spacer grafts suggest that a no-graft technique may be preferable.

**Level of Evidence III:**

This journal requires that authors assign a level of evidence to each article. For a full description of these Evidence-Based Medicine ratings, please refer to the Table of Contents or the online Instructions to Authors www.springer.com/00266.

## Introduction

Lower eyelid retraction is a common eyelid malposition in which the lower eyelid is displaced inferiorly with varying amounts of lateral canthal tendon laxity. Clinically, it causes exposure of the sclera between the limbus and eyelid margin, and symptoms vary from mild irritation and discomfort to vision-threatening corneal exposure and decompensation. Treatment is based on the etiology of the eyelid retraction as well as the degree of symptoms. A variety of treatments for lower eyelid retraction have been described, ranging from supportive medical management (e.g., lubrication) to non-surgical methods (hyaluronic acid filler injection) to surgical techniques that include temporary and permanent tarsorrhaphy, anterior lamellar full thickness skin graft, lower eyelid retractor disinsertion and midface lift with or without the use of a spacer graft.

Spacer grafts are used in lower eyelid retraction repair to increase the vertical dimension of the middle and posterior lamellae in cases of ‘negative vector’ where the globe projects anteriorly from the inferior orbital rim. Several different graft materials have been used, including tarsoconjunctiva, sclera, hard palate, cartilage, buccal membrane, fascia, synthetic devices and bioengineered acellular dermal matrix (AlloDerm, LifeCell Corporation, The Woodlands, Texas). Each method of repair has advantages and disadvantages, and controversy exists about the optimal technique. While some studies have found the use of a graft to enhance surgical outcomes [1], the type of graft does not appear to influence results of lower eyelid retraction repair.[2]

Two commonly used graft materials are hard palate and acellular dermal matrix. Hard palate grafts are composed of a firmer tissue that more closely mimics the tarsus, and already have a mucous membrane to mimic the conjunctiva and offer the advantage of being autologous.[3] AlloDerm grafts possess low complication rates and do not require a donor site; however, they have less structural stiffness than some other graft types and also have a higher tendency to contract and resorb.[4] Prior studies have compared midface lift with hard palate graft to no-graft techniques [1] and midface lift with hard palate graft versus acellular dermal matrix graft techniques.[3]

To the best of our knowledge, no studies compared midface lift for lower eyelid retraction repair performed in conjunction with either retractor disinsertion, no graft, hard palate graft or AlloDerm graft.

## Methods

After obtaining approval from the Cleveland Clinic’s Institutional Review Board, the charts of patients who underwent lower eyelid retraction repair for all etiologies performed by a single surgeon (JDP) at the Cole Eye Institute were reviewed. Four groups were defined: lower eyelid retraction repair with subperiosteal midface lift and 1. hard palate autologous graft (HPG), 2. acellular cadaveric AlloDerm graft (ADG), 3. lower eyelid retractor disinsertion (RD) and 4. no spacer graft or retractor disinsertion (NG). Starting in July 2020 and working in reverse chronological order, charts were reviewed until 30 patients were identified for each group.

Inclusion criteria were patients who underwent lower eyelid retraction repair with at least 4 weeks of follow-up and pre- and postoperative photographs taken with neutral gaze in a frontal view. Exclusion criteria included any of the following: less than 4 weeks of follow-up, patients undergoing any other concomitant surgery or patients without adequate quality photographs (i.e., blurry, low resolution, non-neutral gaze, non-frontal view).

The main outcome measure was the amount of eyelid elevation, calculated as preoperative MRD2 minus postoperative MRD2. Additional outcome measures included surgical and postoperative complications, need for re-operation and improvement in signs of exposure keratopathy.

**Fig. 1:**
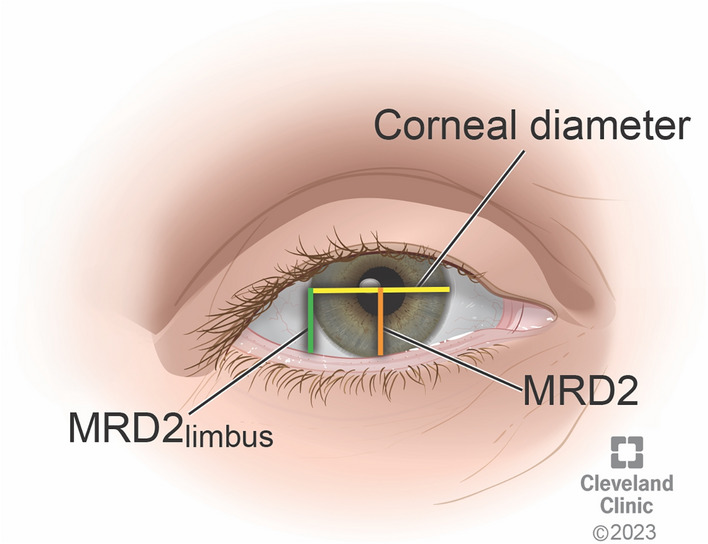
Representative schematic demonstrating measurements taken from preoperative and postoperative patient photographs. Corneal diameter was measured as the distance between the borders of the corneal limbus. MRD2 (marginal reflex distance) was measured from the pupil center to the lower lid margin. MRD2_limbus_ was measured from the lateral limbus at midpupillary line to the lower margin

Measurements of corneal diameter, MRD2 (marginal reflex distance, distance from pupil center to lower lid margin) and MRD2_limbus_ (distance from lateral limbus to lower lid margin) in pixels were obtained directly from patient charts using image analysis tools in Epic EMR software (Figure 1). Measurements were then converted to mm by normalizing the ratio of MRD2 or MRD2_limbus_ to corneal diameter in pixels to a standard corneal diameter of 11 mm as previously described.[5] In all cases, measurements from preoperative photographs were compared to postoperative photographs from the date of latest follow-up to determine amount (in mm) of MRD2 and MRD2_limbus_ elevation achieved.

The decision of which surgical technique to use was based on surgeon preference. Cheeklift was performed in similar fashion for each type of graft. After performing a lateral canthotomy and cantholysis, the conjunctiva and lower eyelid retractors are incised just inferior to the inferior tarsal border across the entire extent of the lower eyelid to the caruncle. The dissection was continued in the pre-septal plane to the level of the arcus marginalis. The arcus marginalis was incised 1-2 mm inferior to the inferior orbital rim and the periosteum widely reflected from the face of the maxilla and zygoma, limited medially by the infraorbital neurovascular bundle, inferiorly by the buccal sulcus and laterally by the masseteric fibers. The periosteum was incised inferiorly in order to obtain wide mobility of the entire cheek, and the myocutaneous flap was suspended superiorly with of 4-0 PDS suture from the suborbicularis oculi fat (SOOF) tissue to the superior cuff of periosteum.

For graft placement, the same procedure was followed for both hard palate buccal mucosal graft and AlloDerm. The inferior palpebral conjunctiva was undermined until mobilized, and the graft is sutured to the inferior tarsal border with running 6-0 plain gut suture. The inferior border of the graft was then sutured to the superior edge of the free conjunctiva in a lamellar fashion. In the group without a graft, no graft was placed.

In the retractor disinsertion group, after mobilization of the inferior palpebral conjunctiva, the lower eyelid retractors were sharply disinserted from conjunctiva for 4-6mm across the entire horizontal extent of the lower eyelid into the cul-de-sac and excised. The remaining lower eyelid retractors were allowed to recess into the fornix in graded fashion, and the conjunctiva was not re-sutured to the inferior border of tarsus.

In all groups, the lateral lower eyelid was shortened horizontally to the appropriate tension and resuspended to the inner aspect of the lateral orbital rim periosteum. All groups also underwent placement of a Frost suture at the end of the case, suspending the lower eyelid through the upper eyelid to the eyebrow, which was left in place for 1 week post operatively. A pressure patch was placed in all cases.

A Kruskal–Wallis test was used to compare changes in MRD2 and MRD2_limbus_ between the four treatment groups, and descriptive statistics are reported as median with interquartile range (IQR). Secondary outcomes including reoperation rates, major and minor complications, and patient satisfaction with symptom and keratopathy improvement were also recorded. Differences in number of reoperations, major complications and minor complications between groups were assessed using Fisher’s exact test. Statistical significance was defined as p<0.05 for all analyses.

## Results

**Fig. 2:**
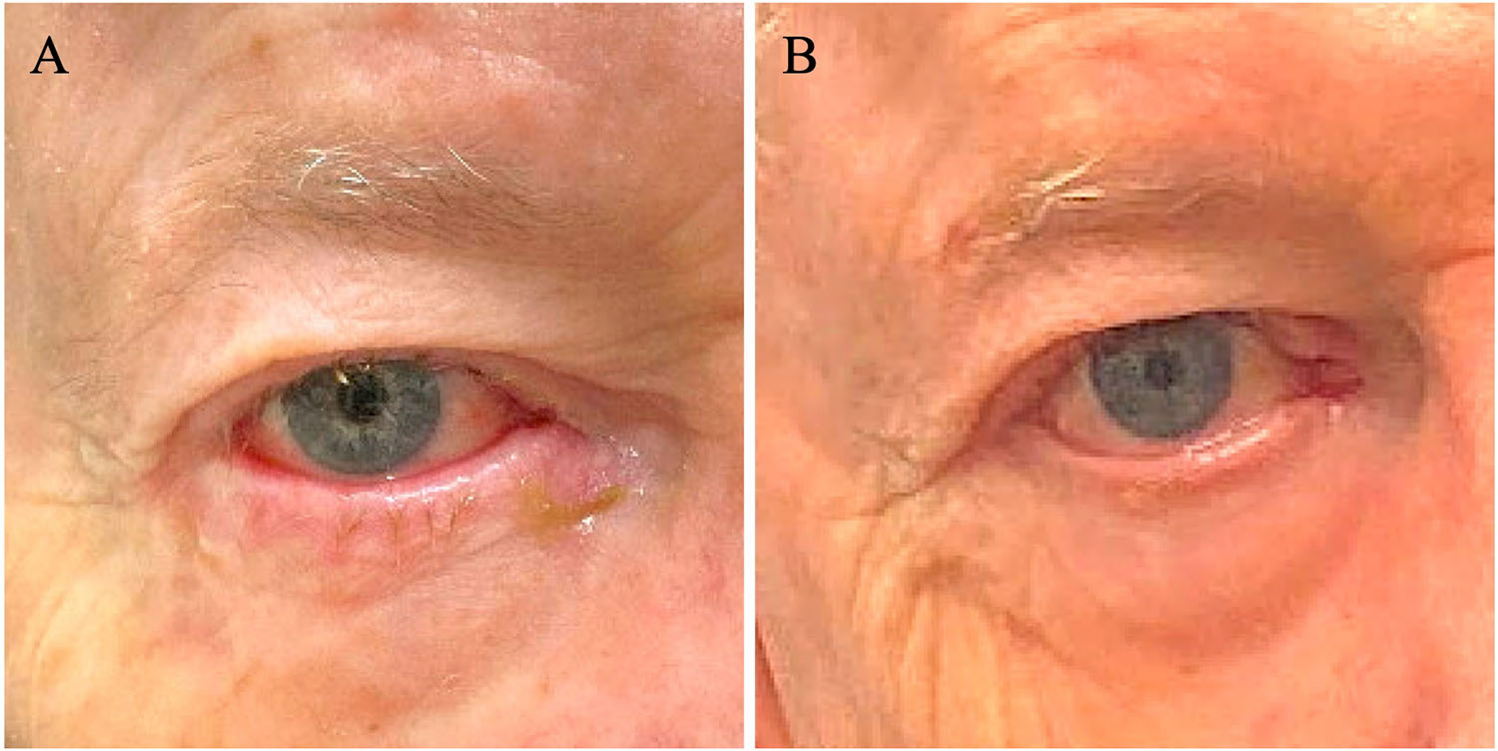
Clinical appearance of a patient with right epiphora. **A** Preoperative photograph demonstrating right lower lid cicatricial ectropion, right lower punctal ectropion and erythema of the lid margin. **B** Postoperative photograph 6 weeks after right cheeklift without graft (NG). Note resolution of the conjunctival injection and right lower lid erythema

**Fig. 3:**
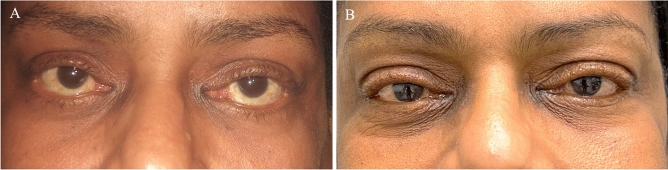
Clinical presentation of a patient with bilateral lower eyelid retraction and exposure keratoconjunctivitis. **A** Preoperative appearance of the patient showing bilateral lower eyelid ectropion, retraction and inferior scleral show. **B** Postoperative photograph 5 years after bilateral cheeklift with retractor disinsertion (RD) demonstrates improvement in margin to reflex distance 2

Twenty-nine patients were excluded from NG analysis (27 for no photographs on file, 2 for poor-quality photographs), 17 from HPG (12 for no photographs, 2 for poor-quality photographs), 17 from ADG (no photographs) and 8 from RD (no photographs). Analysis was performed on 120 procedures (eyelids) from 88 patients meeting inclusion criteria. Twenty-four patients (30 eyelids, 16 females and 14 males) underwent midface lift with no internal spacer graft material (NG) (Figure 2), 23 patients (30 eyelids, 16 females and 14 males) midface lift with hard palate graft (HPG), 19 patients (30 eyelids, 17 females and 13 males) midface lift with AlloDerm graft (ADG), and 22 patients (30 eyelids, 19 females and 11 males) underwent retractor disinsertion (RD) (Figure 3). The median patient age for all procedures was 72 years (IQR, 62 – 81 years) (Table 1). Patients in the NG group underwent surgery from Feb 2013 to July 2020, HPG from April 2013 to April 2016, ADG from July 2013 to Aug 2019 and RD from May 2017 to July 2020. The average follow-up time for all procedures was 20 weeks (range, 4–347 weeks).

**Table 1:** Patient characteristics in 4 different surgical techniques for lower lid retraction malposition repair.

	No graft (*n *= 30)	Hard palate graft (*n *= 30)	AlloDerm graft (*n *= 30)	Retractor disinsertion (*n *= 30)
Female [*n*(%)]	16 (53%)	16 (53%)	17 (57%)	19 (63%)
Age in years [median (IQR)]	83 (78–86)	61 (56–66)	74 (64–80)	69 (65–76)
Average follow-up time (weeks)	14	30	19	16

**Fig. 4:**
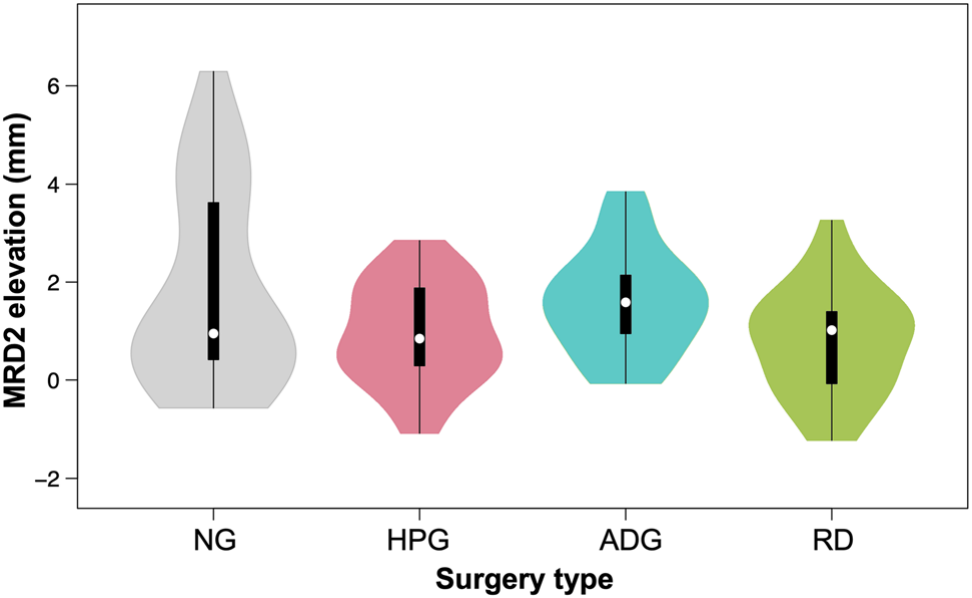
Comparison of MRD2 elevation in 4 different surgical techniques for lower lid retraction repair. Measurements from pre- and postoperative photographs were compared to determine the amount (in mm) of MRD2 (marginal reflex distance, from pupil center to lower lid margin) elevation achieved. Data distributions are represented as violin plots, with median and interquartile range represented by a white dot and thick black bar, respectively. *NG* No spacer graft, *HPG* hard palate graft, *ADG* AlloDerm graft, *RD* retractor disinsertion

**Fig. 5:**
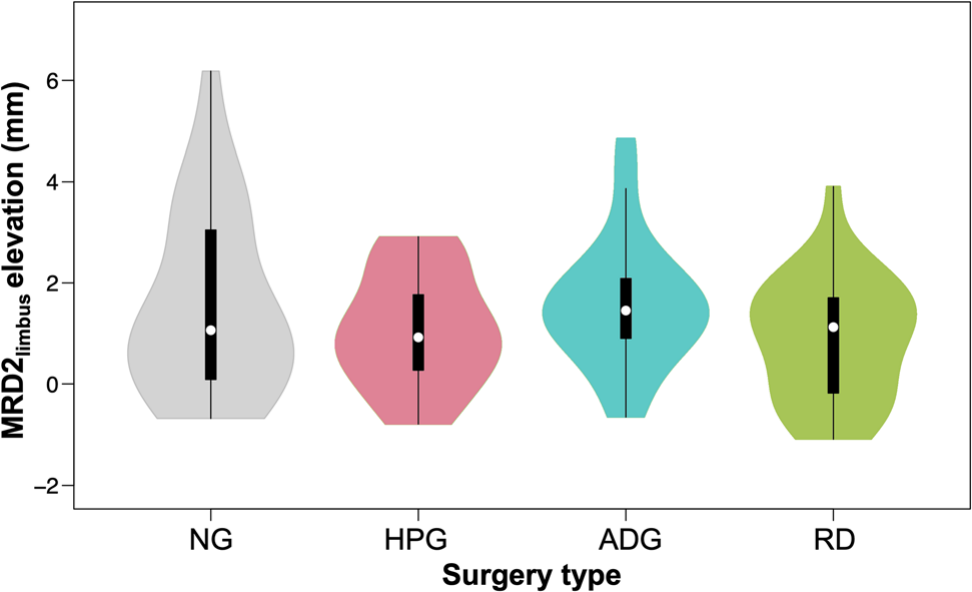
Comparison of MRD2_limbus_ elevation in 4 different surgical techniques for lower lid retraction repair. Measurements from pre- and postoperative photographs were compared to determine the amount (in mm) of MRD2_limbus_ (limbus marginal reflex distance, from lateral limbus at midpupillary line to the lower lid margin) elevation achieved. Data distributions are represented as violin plots, with median and interquartile range represented by a white dot and thick black bar, respectively. *NG* No spacer graft, *HPG* hard palate graft, *ADG* AlloDerm graft, *RD* retractor disinsertion

Symptom/keratopathy improvement was noted in patient charts for 95/92% (NG), 59/70% (HPG), 100/100% (ADG) and 86%/78% (RD) of operations (Table 2). The median MRD2 elevation achieved by each surgery was 0.95 mm (NG), 0.85 mm (HPG), 1.59 mm (ADG) and 1.02 mm (RD) (Figure 4). There were no statistically significant differences in MRD2 position changes between the 4 groups (*p* = 0.06). The median MRD2_limbus_ elevation was 1.06 mm (NG), 0.92 mm (HPG), 1.45 mm (ADG) and 1.12 mm (RD) (Figure 5). There were no statistically significant differences in MRD2_limbus_ position changes between the 4 groups (*p* = 0.29) (Table 3).

**Table 2:** Patients with improvement in keratopathy signs and symptoms for each of the 4 different surgical techniques for lower lid malposition repair.

	ADG				HPG				NG				RD				All			
	N	X	Y	All	N	X	Y	All	N	X	Y	All	N	X	Y	All	N	X	Y	All
Symptoms improved? (Y/N/X, X=not recorded)	0	4	26	30	7	11	12	30	1	5	24	30	4	2	24	30	12	22	86	120
Keratopathy/signs improved? (Y/N/X, X=not recorded)	0	11	19	30	3	18	9	30	1	18	11	30	5	7	18	30	9	54	57	120

**Table 3:** Postoperative outcomes of 4 different surgical techniques for lower lid malposition repair.

	No graft (*n *= 30)	Hard palate graft (*n *= 30)	AlloDerm graft (*n *= 30)	Retractor disinsertion (*n *= 30)	*p*-value
MRD2 elevation [median (IQR)]	0.95 mm (0.36–3.8)	0.85 mm (0.26–1.95)	1.59 mm (0.90–2.24)	1.02 mm (− 0.09-1.47)	0.06
MRD2_limbus_ elevation [median (IQR)]	1.06 mm (0.03–3.19)	0.92 mm (0.24–1.95)	1.45 mm (0.86-2.11)	1.12 mm (− 0.21-1.77)	0.29
Major/minor complications (*n*)	1/5	2/5	1/5	1/3	1.00/0.871
Reoperations [*n*(%)]	2 (7%)	10 (33%)	3 (10%)	0 (0%)	0.0006

Major complications included worsening of vision (HPG, *n *= 1), prolapsing graft (HPG, *n *= 1), ectropion (NG, RD; *n *= 1 for each) and lid retraction (ADG, *n *= 1) (Table 3). Minor complications occurred in 5 NG, 5 HPG, 5 ADG and 3 RD operations (Table 3) and included symptoms such as pain, tearing, discharge and suture granuloma formation. Reoperation rates significantly differed between techniques and were highest with the use of hard palate graft (33%) followed by AlloDerm graft (10%), no graft (7%) and retractor disinsertion (0%) (*p* = 0.0006, Table 3). The most common reoperation procedure for hard palate graft recipients was tarsorrhaphy (*n *= 6). Other reoperations for this group included lateral canthal repositioning after a prolapsed graft, cosmetic lower blepharoplasty, external levator resection and conjunctivoplasty (*n *= 1 for each). Of those receiving an AlloDerm graft, reoperations included tarsorrhaphy (*n *= 2) and cosmetic lower blepharoplasty (*n *= 1). Both reoperations for those with no graft were retractor disinsertions (*n *= 2). Retractor disinsertion technique had 0 reoperations.

## Discussion

There is a wide body of the literature describing satisfactory results obtained from the use of new materials and comparing spacer graft materials such as AlloDerm vs. hard palate graft (for a comprehensive review, see [4]). Recently, others have shown that the use of no spacer graft can also achieve excellent lower lid elevation with low complication rates.[5] However, to our knowledge no current studies have directly compared results obtained from no spacer graft with the use of hard palate and AlloDerm grafts from a single surgeon. This approach allows for factors such as surgical technique and data measurements to be maintained constant for each procedure, thus making the results from each surgical group more directly comparable.

Based on the results of our study and previously published case series [1, 3], we had anticipated that the hard palate graft would provide the greatest amount of lower eyelid lengthening, followed by the AlloDerm graft. Interestingly, we found no significant difference between any of the techniques. It is possible that the underlying mechanism for improvement in all 4 surgical techniques results from recruiting anterior lamellar tissues via the midface lift, rather than in distancing the eyelid retractors from the tarsus or to the graft itself lengthening the posterior lamella. The lack of statistically significant difference in MRD2 between groups using HPG and no graft has been attributed in another study to a small sample size.[3] Another prospective, randomized trial comparing different spacer graft materials was unable to ascertain statistical significance, attributed to sample size, short follow-up times and lack of matching for age, gender and etiology of retraction [2]. It appears that the addition of a posterior lamellar graft to the midface lift does not augment results in terms of ability to elevate the lower eyelid margin against the cornea.

This is a salient finding, since the use of spacer grafts was also found to be associated with higher reoperation rates. Hard palate grafts in particular were associated with the highest reoperation rates and the least amount of symptom and keratopathy improvement when compared to the other techniques evaluated. By eliminating the necessity for auto- and allograft material, there is less potential for complications such as infection at the donor site or graft resorption. It may also be more cost and time effective to forego the implantation of graft material.

This study suffers from significant limitations. First, the patient population was not homogeneous, and the etiology of lower eyelid malposition varied. These included causes such as postblepharoplasty lower eyelid retraction, thyroid eye disease, trauma, congenital and age-related changes. This could make the applicability to specific etiologies difficult to interpret. In the future, a prospective study design would allow categorization of etiologies of eyelid retraction and objective documentation of keratopathy signs and symptoms. However, the major strength of this study nearly obviates this issue. Each technique was used almost exclusively for a given time period in the overall study period, so the results represent almost a comparison of four different consecutive series. Because of this, it can be inferred that each study group contained similar numbers of the various etiologies of eyelid retraction. Further, groups were relatively balanced in terms of sex ratio and age, further implying similarity between the groups. The median preoperative MRD2 and MRD2_limbus_ also did not vary significantly between each surgical group (*p* = 0.76 and 0.39, respectively). The groups differed in average length of follow-up time, and it is possible that the higher reoperation rates seen in HPG surgery are a consequence of longer monitoring time, which could be addressed as data from patients with more recent surgeries become available.

Notably, there was a shift in the surgeon’s practice patterns during the study period of 7 years. Early in the study period, the surgeon approached ectropion via the use of hard palate grafts, later favoring the use of AlloDerm grafts, and in the latter years, retraction disinsertion was more frequently employed. The surgeon’s heightened experience over time could have allowed improved surgical technique, likely translating to overall improved outcomes in the latest treated patients within the study population. This is beneficial in the interpretation of the data, since it can be inferred that each study group represents a homogenous group in terms of severity of disease. Generally, the surgical approach varied depending on the severity of ectropion, with mild ectropion being treated with retractor disinsertion, moderate ectropion with AlloDerm graft and severe ectropion with hard palate graft.

In conclusion, midface lift alone may elevate the lower eyelid as well as techniques that require a second surgical site (hard palate graft), use of human donor tissue (acellular dermal graft) and techniques that require more extensive tissue manipulation (retractor disinsertion). It is possible that posterior lamellar grafting helps certain types of lower eyelid retraction more than others, and further study regarding specific etiologies may elucidate this question. The senior author currently does not use any of the three more invasive techniques for the vast majority of functional midface lifts to treat lower eyelid retraction.
